# “Call a Teenager… That’s What I Do!” - Grandchildren Help Older Adults Use New Technologies: Qualitative Study

**DOI:** 10.2196/13713

**Published:** 2019-06-06

**Authors:** Jennifer Dickman Portz, Christine Fruhauf, Sheana Bull, Rebecca S Boxer, David B Bekelman, Alejandra Casillas, Kathy Gleason, Elizabeth A Bayliss

**Affiliations:** 1 Division of General Internal Medicine University of Colorado Aurora, CO United States; 2 Institute for Health Research Kaiser Permanente Colorado Aurora, CO United States; 3 Department of Human Development and Family Studies College of Health and Human Sciences Colorado State University Fort Collins, CO United States; 4 mHealth Impact Lab Colorado School of Public Health University of Colorado Aurora, CO United States; 5 Department of Medicine Eastern Colorado Health Care System Department of Veterans Affairs Denver, CO United States; 6 Division of General Internal Medicine and Health Services Research David Geffen School of Medicine University of California, Los Angeles Los Angeles, CA United States

**Keywords:** health technology, eHealth, family research, social support

## Abstract

**Background:**

Although family technical support seems intuitive, there is very little research exploring this topic.

**Objective:**

The objective of this study was to conduct a subanalysis of data collected from a large-scale qualitative project regarding older adults’ experiences in using health information technology. Specifically, the subanalysis explored older adults’ experiences with technology support from family members to inform strategies for promoting older adults’ engagement with new health technologies. Although the primary analysis of the original study was theoretically driven, this paper reports results from an inductive, open-coding analysis.

**Methods:**

This is a subanalysis of a major code identified unexpectedly from a qualitative study investigating older adults’ use experience of a widespread health technology, the patient portal. A total of 24 older patients (≥65 years) with multiple chronic conditions (Charlson Comorbidity Index >2) participated in focus groups conducted at the patients’ primary clinic. While conducting the primary theoretically driven analysis, coders utilized an open-coding approach to ensure important ideas not reflected in the theoretical code book were captured. Open coding resulted in 1 code: *family support*. This subanalysis further categorized *family support* by who provided tech support, how tech support was offered, and the opinions of older participants about receiving family tech support.

**Results:**

The participants were not specifically asked about family support, yet themes around family assistance and encouragement for technology emerged from every focus group. Participants repeatedly mentioned that they called their grandchildren and adult children if they needed help with technology. Participants also reported that family members experienced difficulty when teaching technology use. Family members struggled to explain simple technology tasks and were frustrated by the slow teaching process.

**Conclusions:**

The results suggest that older adults ask their family members, particularly grandchildren, to support them in the use of new technologies. However, family may experience difficulties in providing this support. Older adults will be increasingly expected to use health technologies, and family members may help with tech support. Providers and health systems should consider potential family support and engagement strategies to foster adoption and use among older patients.

## Introduction

### Background

Health care consumers are increasingly *going digital*, and older adults are the fastest growing users of the internet for health information [1]. Health technologies are positively associated with better medical outcomes [2] and have the potential to help older adults live independently, manage their health, and improve communication [3,4]. With increased access to and availability of technological tools, older adults are progressively expected to use emerging health technologies by health care providers and systems.

Older populations face barriers toward the adoption of health technology [4-6], and adoption rates of technology-based interventions remain low [7,8]. During health technology trials, many participants never use the technology available to them, and those who adopt the technology, commonly use the tool only a few times. However, once enrolled in a trial, older adults are more likely to complete a health technology intervention to manage their health than younger patients [9]. The initial adoption of health technologies is key to successful ongoing use.

Many factors contribute to older adults’ adoption of new technologies, including cost, education, perceptions of the technology, human indicators, and health status [10]. The idea that family and friends may have an important role for technology adoption among older adults is fairly new [11]. There is evidence to suggest that older adults prefer learning technology skills from their informal networks, including children, grandchildren, and neighbors [12], and they particularly enjoy using technologies that improve communication with these networks [13]. However, little is known about the experience of the families providing technical support to their older adult loved ones.

### Objectives

The purpose of this subanalysis was to explore older adults’ experiences with technology support from family members to inform strategies for promoting adoption of new health technologies by older adults. Our primary study focused on assessing the user interface (UI) and user experience (UX) of a specific health technology linking patients to their electronic health record—patient portals. To support user-centered design of portal systems, the primary study employed a deductive theoretically driven data analysis strategy, and the results are reported elsewhere [14]. However, our inductive subanalysis of the primary data revealed the fundamental importance of family members in the adoption and use of technology. *Family support* was the only inductive code identified from the data. As the primary study focused on the UI/UX design of the patient portal and not technical support, the *family support* code was further analyzed separately in this subanalysis. The themes of the subanalysis are presented in this paper.

## Methods

### Summary of Primary Study Procedures

The larger study used a qualitative descriptive approach that resulted in the identification of the *family support* code further examined in this subanalysis. All research procedures were approved by the health systems’ institutional review board.

Using the health system’s electronic health record, we randomly identified (N=225) older patients (≥65 years) with multiple chronic conditions (Charlson Comorbidity Index >2). Patients who were non-English speakers, diagnosed with dementia, or residing in a skilled nursing facility were excluded from participation. Potential participants were mailed a letter inviting them to participate in a focus group that included an opt-out phone number. We called (N=210) patients who did not opt-out via phone to schedule. Of the 37 participants who were scheduled for focus groups, 24 participated in the study.

A total of 6 semistructured focus groups were conducted (by JDP). The focus group discussions lasted approximately 90 min and took place at the patient’s primary health clinic. Questions were specific to the primary aim of the study and inquired about function, ease of use, and usefulness of the patient portal website and features. Focus groups were audio-recorded and professionally transcribed for accuracy.

Furthermore, 2 doctoral trained researchers conducted the analysis (JDP: primary investigator and KG: research assistant). The primary analysis used a theoretically driven code book founded in the technology acceptance model [15] related specifically to usability and use experience of the portal system. However, during initial coding, coders also used *open coding,* an inductive approach [16], to capture potentially meaningful information from responses.

### Subanalysis of Family Support Code

After reviewing the first cycle codes, analysts identified that participants in every focus group referred to a family member helping with technology. The coding team labeled these responses *family support*. *Family support* was the only inductive code identified from the data. We then used a *heading and subheading* thematic approach [16] to further investigate the *family support* codes focused on who provided the tech support, how the tech support was offered, and participants’ opinions about receiving *family support* technical assistance. This technique resulted in 3 subthemes reported below.

## Results

### Participants

Overall, 24 patients participated in focus groups (Table 1). Study participants were aged, on average, 78.4 (SD 5.4) years, and 50% were female. All participants were high school graduates, most attended college, and most participants’ income was between US $30,000 and $50,000. All but 1 participant had a cell phone, primarily smartphones. Many participants regularly used email, the internet, computers, and social media.

### Family Support for Using Technology

The subanalysis resulted in 3 subthemes: assistance from grandchildren and adult children, relationship building from technology, and potential challenges with family support. We did not specifically ask about family support, family members helping with the portal, or experiences working with family members to use technology. However, the inductive coding process revealed that family members were helping older adults to adopt and use new technologies, and grandchildren were the most commonly discussed. A few participants noted help from adult children as well. Participants were eager to share stories about their grandchildren and were impressed by their grandchildren’s innate abilities to use technology. Responses suggested that participants experienced relationship building with their family members from learning new technological skills and using technologies to communicate. Although they were excited to seek help from grandchildren to use phones, televisions, or computers, the participants also identified challenges to obtaining help. Participants reported that grandchildren and adult children had a difficult time slowing down and explaining tasks to their parent or grandparent. Participants were also concerned that their grandchildren could break or further complicate the technology, that is, “mess up the remote-control settings.” Participant quotes, representing participants from all focus groups, related to these findings are summarized in Table 2.

**Table 1:** Participant characteristics (N=24).

Participant characteristic		Value
Age, mean (SD)		78.4 (5.4)
Female, n		12
White, n		19
Hispanic, n		3
**Education, n**		
	High school graduate	6
	Some college	9
	College graduate	9
**Income, n**		
	<US $30,000	4
	US $30,000-49,999	13
	US $50,000-74,999	2
	>US $75,000+	2
	Choose not to answer	3
**Own cell phone, n**		
	Smartphone	17
	A regular or basic phone	6
	Does not have a cell phone	1
**Regular technology utilization, n**		
	Email	22
	Look up information online	21
	Use social media	13
	Play computer games	15
	Video chat	11
	Instant messaging	8

**Table 2:** Family support quotations.

Subtheme	Quote
Assistance from grandchildren and adult children	“My grandkids do that. I mean, like I said, if I have a problem, I call my grandkids. They're teenagers.” “I give [Mia] the phone and she just zips right through it. Okay, thank you, I'm where I can work it now. Bye.”
Relationship building from technology	“Well, my son's forty—let's see, my daughter's fifty. He'll be forty-eight this year, so he's always been a computer geek. So he builds [computers] and all that kind of crud. If I'm having a problem, I call him.” “I think I still have my original flip phone, but my kids said, mom, you need this [smart phone].” “It’s like whenever I don’t know something, I ask [my grandchildren], and they know. So it’s pretty cool. And then it’s kind of cool because I get to learn all the new lingo and that sort of thing.” “Facebook, I got the app on there and my granddaughter helped me a little bit. She lives in California, so I don’t see her very often.” “I use computer somewhat for email and stuff and then when I got my iPhone, I abandoned the computer. I may go on it once a month because I do Facebook and email. I Facetime with my granddaughter…I do!” “I use Facebook. That's how I keep track of my daughter and grandson and granddaughter.”
Potential challenges with family support	“She will sometimes slow down and have the patience to teach me. ‘Granny, you know.’ She just gets frustrated with me because it comes slow to me…She is just a wiz on that thing.” “There are certain things that my son is going to teach me how to do something, and it’s so instinctive to him that he doesn’t even know how to explain it.” “But you want to also be careful of 5- and 6-year-olds, because they could screw everything up, they really could. The reason they get something done is because they’re not afraid to try.”

## Discussion

### Principal Findings

Family support may have a key role in the successful adoption and use of emerging health technologies [17]. Our participants suggest that grandchildren and adult children are helping their (grand)parents learn to use new technology, troubleshoot issues, and adapt new technologies to older adults. This supports recent studies suggesting that children and grandchildren help older family members in the uptake of technology, purchasing devices, and installing equipment [18]. Grandchildren specifically were found to be the primary reason for older adults’ initial tablet use [19]. In a recent mobile health (mHealth) project, older adults specified children and grandchildren as their primary tech support contact and preferred using family over online manuals [20]. As older adults are seeking assistance from their informal network, providers and health systems should consider family support engagement as potential strategies to foster adoption and use of health technology among older patients.

Results from the focus groups also indicate that family members encounter some challenges in teaching new technology skills. This is contrary to previous research that suggests that grandchildren proudly teach their grandparents how to use electronic devices [21]. Another study also found that grandchildren were excited to teach their grandparents how to use Nintendo Wii, a gaming console, for exercise purposes [22]. Regardless, adult children and grandchildren may benefit from assistance or tips for how to navigate these frustrations while helping their older family members [17].

Additional research identifies that when children and grandchildren are involved with tech support, older adults are less likely to *play* or *figure out* how to use and fix technology, as they will wait for their family to solve the tech issue [18]. Peek et al [23] also found that older adults are sometimes afraid to burden their children and family with technology needs. Family members can only help to an extent with specific technologies. Manuals and tech support will likely remain important elements to support health technology adoption among older adults [24].

We did not elicit specific information about family support, yet the participants regularly documented the importance of their family members. The process of learning to use new technologies and using communication tools, such as Facetime and Facebook, connected our participants with their family. Currently, most health technologies are designed for an individual user, either a patient or caregiver, or to monitor an older adult providing specific information to a caregiver. Designing health technology systems with a creative family approach, rather than a single user, may improve adoption, use, and ultimately, health outcomes. For example, researchers recently examined a grandparent-grandchild mobile Health Buddies app to promote health knowledge, and it was found acceptable to use by participants [25].

### Limitations

As a subanalysis, there are several limitations to our work. Our primary focus of the larger study was not to identify family support. We did not ask follow-up questions or inquire about family support experiences; therefore, we lack a full understanding of this type of technology assistance. We were able to conduct 6 focus groups with 24 participants, indicating saturation of our themes may not have been fully accomplished. We were unable to capture specific strategies used by grandchildren and adult children to help their older family members. Participants were mostly white and well educated with health coverage; thus, the experiences of family support are limited to this case study. Not all older adults have adult children or grandchildren, and the results did not capture other relevant forms of social support.

### Conclusions

There is growing evidence to suggest that families assist older adults in the adoption of new health technologies. This study proposes that older adults are specifically reaching out to their adult children and grandchildren. Although family technology support appears beneficial, there may be some challenges for older adults and their family members. On the basis of our unexpected findings related to family technical assistance, it is important to consider the family context and include family members in the implementation of new health technology as they are likely helping older users.

## Abbreviations

mHealth: mobile health
UI: user interface
UX: user experience
